# High level of hemoglobin, white blood cells and obesity among Sudanese women in early pregnancy: a cross-sectional study

**DOI:** 10.4155/fsoa-2016-0096

**Published:** 2017-04-04

**Authors:** Abdelmageed Elmugabil, Duria A Rayis, Renda E Abdelmageed, Ishag Adam, Gasim I Gasim

**Affiliations:** 1 Faculty of Medicine, El Imam El Mahdi University, Kosti, Sudan; 2 Faculty of Medicine, University of Khartoum, P.O. Box 102, 11111, Khartoum, Sudan; 3 Faculty of Medicine, Alneelain University, Khartoum, Sudan

**Keywords:** hemoglobin, obesity, pregnancy, Sudan, white blood cells

## Abstract

**Aim:**

To investigate the association between obesity and anemia/hemoglobin levels.

**Material & methods:**

A cross-sectional study was conducted at Khartoum, Sudan. Obstetric data were collected from 388 pregnant women at mean (standard deviation) of 10.5 (3.1) weeks of gestation using questionnaires. Weight and height were determined, and BMI was calculated.

**Results:**

There were 15 (4.4%), 95 (28.1%), 127 (37.6) and 101 (29.9%) women who were underweight, normal weight, (18.5–24.9 kg/m^2^), overweight (25–29.9 kg/m^2^) and obese (≥30 kg/m^2^), respectively. Hemoglobin levels and white blood cell counts were significantly higher in obese than nonobese groups. Compared with normal BMI, overweight and obesity were associated with higher hemoglobin level.

**Conclusion:**

Obese women had higher white blood cell count and hemoglobin level.

## Lay abstract

Obesity during pregnancy has many maternal and perinatal adverse effects. The current study was conducted to investigate the association between obesity, hemoglobin level and white blood cell count among pregnant Sudanese women in their early pregnancy. Around a third of the 388 women were obese. Hemoglobin level and white blood cells count were significantly higher in obese than nonobese women. Further studies are needed to fully understand the implications of this relationship.

## Background

The epidemic of obesity is a significant public health problem worldwide, the complications and co-morbidities of which can result in chronic diseases among adults and children [1]. Pregnant women are not protected against this trend among the general population: obstetricians and midwives report seeing an increasing number of obese pregnant women, with prevalence rates around 20% detected [2]. The situation among African pregnant women, where pregnancy obesity rates have reached very high levels (approaching 50%), is far more complicated [3,4]. An association between obesity and hemoglobin levels has long been assumed [5,6]. Driven by the current obesity epidemic worldwide, numerous studies have been conducted into the link between obesity and anemia (and/or changes in hemoglobin levels) among the general population [7–9]. Despite this, little research has been done into the same issue during pregnancy. Classical obesity-associated anemia is characterized by dietary iron deficiency, a higher need for iron because of increased blood volume, physical inactivity and low-grade inflammation [7–9]. As part of the bodies’ physiological adaptation and preparation for pregnancy, the total blood volume experiences an increase of about 1.5 l, in order to meet the demands of the new vascular bed and to anticipate the blood loss occurring at delivery [10]. Along with this, the red cell mass (stimulated by the step up of the maternal erythropoietin production) also increases, however, to a relatively less extent, when compared with the increase in plasma volume, with an overall result being a drop in hemoglobin concentration, resulting in dilutional anemia.

Anemia during pregnancy has been defined by the WHO as a hemoglobin of <11 g/dl [11]. It typically presents with weakness, fatigue, dyspnea and exercise intolerance, a presentation that can significantly preclude weight loss in obese subjects. Likewise, anemia is known to increase the risk of heart failure and mortality [8,12]. Recently, hepcidin (an iron-regulatory protein) has been the target of research, as it is thought to play a key role – along with obesity-associated low-grade inflammation – in the regulation of iron homeostasis [13–16]. However, as there is still a lack of consensus about the association between obesity and anemia [17–20], it is unclear whether obesity itself is a risk factor for developing anemia.

Obesity in pregnancy is associated with unfavorable pregnancy outcomes [21], along with increased risk of delivery via cesarean section and higher neonatal intensive care admissions [22,23]. These challenging repercussions, and their associated health costs [24], mandate further research into this global problem. Here we investigated the assumed link between obesity and anemia/hemoglobin levels among a population of Sudanese women during early pregnancy.

## Materials & methods

### Methods

A cross-sectional study was conducted at Saad Abualila Hospital (Khartoum, Sudan) during the period of January–April 2014. This is a tertiary semiprivate hospital governed by the Faculty of Medicine, University of Khartoum. After giving informed consent, eligible women were enrolled in the study. Inclusion criteria were: early pregnancy (<14 weeks of gestation), singleton pregnancy and willingness to participate in the study. Iron supplement was not yet started and was not recommended in the first trimester. Women with diabetes mellitus, hypertension or any other chronic disease were excluded from the study. A trained medical officer used a questionnaire to gather data from each pregnant women on her age, parity, educational level (illiterate, primary school education or secondary school and above education), occupation (housewife or working mother), gestational age calculated in weeks (based on last menstrual cycle and confirmed by ultrasound) and medical diseases (diabetes, hypertension). Weight and height were determined, and BMI was calculated and expressed as weight in kilograms divided by the square of height in meters. The complete maternal hemogram with blood cell count was measured for all women, using an automated hematology analyzer, as previously described [25–27].

BMI was categorized into four groups: underweight (<18.5 kg/m^2^), normal weight (18.5–24.9 kg/m^2^), overweight (25–29.9 kg/m^2^) and obese (≥30 kg/m^2^) following the WHO which addresses obesity during pregnancy as well as the general population [28].

### Statistics

SPSS for Windows (version 16.0) was used for data analyses. A total sample size of 338 participants was calculated using the previous incidence of obesity (19.4%) among pregnant women in Sudan [3]. A formula was used to calculate the difference in the mean of the proposed variable (hemoglobin level as 11.0 and 10.6 g/dl in the obese and normal weight women, respectively) that would provide 80% power to detect a 5% difference at α = 0.05, with an assumption that complete data might not be available for 10% of participants. One-way analysis of variance (with *post hoc* Bonferroni tests) and χ^2^ tests were used to compare the normally distributed continuous variables and proportions between BMI groups. The variables of the one-way analysis of variance and χ^2^ test were entered (if p < 0.2) in a multinomial logistic regression (stepwise with forward entry) where BMI group was the dependent variable and sociodemographic parameters (age, parity, education, job, residence), hemoglobin levels and white blood cells (WBCs) count were the independent variables. Odds ratio (OR) and 95% CI were calculated, using women with normal weight as the control group. p < 0.05 was considered statistically significant at two-sided test.

### Ethics

The study received ethical clearance from the Research Board at the Department of Obstetrics and Gynecology, Faculty of Medicine, University of Khartoum, Sudan.

## Results

General characteristics of the 338 enrolled women are shown in Table 1. Over half (176, 52.1%) of the women were primiparae. The mean (standard deviation) of the age, parity and gestational age was 27.8 (5.5) years, 0.9 (1.2) and 10.5 (3.1) weeks, respectively. Approximately half (168, 49.7%) of these women were anemic (hemoglobin <11 gm/dl).

**Table 1.** **General characteristics of Sudanese women in the current study in their early pregnancy.**

**Variable**	**n = 338**
**The mean (SD) of**	
Age (years)	27.8 (5.5)
Gravidity	2.3 (1.60
Parity	0.9 (1.2)
Gestational age (weeks)	10.5 (3.1)
Interpregnancy interval (months; n = 160)	38.6 (2.4)
BMI, 18.5 kg/m^2^	27.7 (5.7)
Hemoglobin (gm/dl)	10.8 (1.1)
White blood cell (cells × 10^9^/l):	7740 (2174)
– Neutrophils	5056 (1,820)
– Lymphocytes	2167 (580)
Lymphocytes/neutrophils	0.48 (0.41)
Red cells distribution width (%)	13.8 (1.5)
**Number (%) of**	
Rural residence	48 (14.2)
Education level ≤secondary level	46 (13.6)
Housewives	252 (74.6)
Anemia	168 (49.7)
History of:	
– Miscarriage	73 (21.6)
– Stillbirth	6 (1.8)
– Gestational diabetes	5 (1.5)
– Pre-eclampsia	6 (1.8)

SD: Standard deviation.

Of the 338 enrolled women, 15 (4.4%), 95 (28.1%), 127 (37.6%) and 101 (29.9%) were underweight, normal BMI, overweight and obese, respectively (Table 2).

**Table 2.** **Comparison of sociodemographic, medical and obstetric characteristics between BMI groups among early-pregnancy Sudanese women.**

**Variables**	**Underweight n (15)**	**Normal n (95)**	**Overweight n (127)**	**Obese n (101)**	**p-value**
**The mean (SD)**					
Age (years)	27.0 (6.5)	26.3 (5.6)	28.1 (5.4)	28.9 (5.1)	0.007
Gravidity	2.1 (1.9)	1.7 (1.1)	2.4 (1.7)	2.7 (1.7)	<0.001
Parity	0.5 (0.9)	0.5 (0.8)	1.1 (1.4)	1.3 (1.3)	<0.001
Gestational age (weeks)	10.5 (3.2)	10.3 (3.0)	10.4 (3.0)	10.7 (3.5)	0.816
Interpregnancy interval	48.2 (27.0)	32.3 (21.4)	37.1 (23.1)	42.2 (26.7)	0.225
BMI, 18.5 kg/m^2^	17.2 (0.8)	22.5 (1.7)	27.3 (1.4)	34.5 (4.1)	<0.001
Hemoglobin (gm/dl)	10.1 (1.4)	10.6 (1.0)	11.0 (1.0)	11.0 (1.1)	0.003
White blood cell (cells × 10^9^/l):	6100 (1430)	7446 (1988)	7937 (2104)	8013 (2396)	0.004
– Neutrophils	3788 (1197)	4902 (1590)	5164 (1828)	5253 (2015)	0.022
– Lymphocytes	1983 (656)	2128 (665)	2163 (541)	2236 (527)	0.338
Lymphocytes/neutrophils	0.692 (0.577)	0.514 (0.701)	0.455 (0.164)	0.471 (0.188)	0.181
Red cells distribution width (%)	13.8 (1.6)	13.8 (1.6)	13.7 (1.5)	13.8 (1.6)	0.934
**Number (%) of**					
Rural residence	4 (26.7)	19 (20)	13 (10.2)	12 (11.9)	0.085
Education level ≤secondary level	1 (6.7)	13 (13.7)	16 (12.6)	16 (15.8)	0.764
Housewives	12 (80)	71 (74.7)	95 (74.8)	74 (73.3)	0.955
Anemia	10 (66.7)	56 (58.9)	56 (44.1)	46 (45.5)	0.064

**Figure 1.**
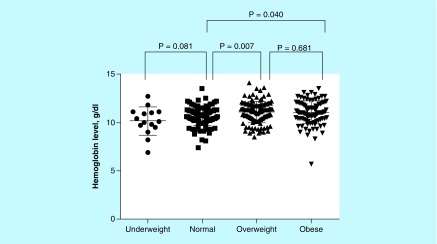
**Relationship between hemoglobin concentration and BMI categories.**

While there was no significant difference in residence, education and occupation, age and parity, and hemoglobin levels were significantly higher in obese than normal groups and they were higher with increasing BMI (Table 2). In comparison with the normal weight group, the mean (standard deviation) of the hemoglobin level was significantly higher in overweight (10.6 [1.0] vs 11.0 [1.0]g, p = 0.007) and obese women (10.6 [1.0] vs 11.0 [1.1]g, p = 0.040), however there was no significant difference in hemoglobin level between the overweight and obese groups (Figure 1).

Interestingly, although reaching borderline significance (p = 0.064), there was no significant difference in the prevalence of anemia between groups.

**Figure 2.**
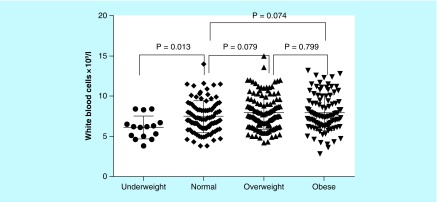
**Relationship between white blood cell count and BMI categories.**

While WBC count was significantly lower in underweight than in normal weight women, there was no significant difference in WBC count between normal weight, overweight and obese women (Figure 2).

In the multinomial analyses, while there was no significant difference in age, residence and educational level between the different BMI groups, parity was higher with increasing BMI (OR [95% CI]: 1.6 [1.1–2.2]; p = 0.003 and 1.7 [1.2–2.4]; p = 0.001 for the overweight and obese women, respectively). In comparison to normal weight, overweight and obesity were associated with higher hemoglobin level (OR: 1.4 [1.1–1.8] and 1.3 [1.06–1.8], respectively). The OR for WBC count in the underweight and obese women compared with normal weight women were 0.6 (0.4–0.9); p = 0.012 and 1.1 (0.9–1.3); p = 0.054, respectively. Neutrophils were only significantly different in the underweight compared with normal weight group (OR: 0.5 [0.3–0.8]; p = 0.013) (Table 3).

**Table 3.** **Multinomial analyses of the factors associated with underweight, overweight and obesity.**

**Variables**	**Underweight (n = 15)**	**Overweight (n = 127)**	**Obese (n = 101)**
Age (years)	1.05 (0.9–1.2); 0.423	1.0 (0.9–1.0); 0.555	1.0 (0.9–1.1); 0.409
Parity^†^	0.7 (0.3–1.5); 0.435	1.6 (1.1–2.2); 0.003	1.7 (1.2–2.4); 0.001
Hemoglobin (gm/dl)	0.6 (0.4–1.0); 0.091	1.4 (1.1–1.8); 0.005	1.3 (1.06–1.8); 0.016
White blood cells	0.6 (0.4–0.9); 0.012	1.1 (0.9–1.2); 0.075	1.1 (0.9–1.3); 0.054
Neutrophils	0.5 (0.3–0.8); 0.013	1.1 (0.9–1.2); 0.210	1.1 (0.9–1.3); 0.125

Data expressed as odds ratio (95% CI); p-value.

^†^Adjusted for age; white blood cells and neutrophils were entered one by one in each model.

## Discussion

A recent meta-analysis showed that the prevalence of maternal obesity across Africa ranged from 6.5 to 50.7% [4]. The current study found a high (29.9%) prevalence of obesity among pregnant women (especially women with high parity) in their early pregnancy independent of their residence, education and occupation. Recently a much lower prevalence (19.4%) of obesity was reported among pregnant women in Khartoum, Sudan [3]. Even though these two studies were conducted in the same setting (Khartoum), socioeconomic and methodological factors could explain this discrepancy. The current study was conducted in a semiprivate hospital, while that of Rayis *et al*. was conducted in a general hospital, possibly reflecting a hidden bias based on socioeconomic factors. Notably, it is difficult (if not impossible) to investigate socioeconomic factors in Sudanese women, as the flow of income between relatives and neighbors common in this culture can confound accurate income assessment. The other difference between the studies was that Rayis *et al*. studied women in their later gestational age while the current study assessed women in their early gestational age.

In the current study, age was associated with obesity in the univariate analyses alone, with no significant association between age and obesity in the multinomial analyses. Therefore, age was considered as a confounder. In the previous report in the same setting, maternal obesity was positively associated with age (OR: 1.1; 95% CI: 1.08–1.2) [2]. Moreover, it has also been reported that obesity increased with increasing maternal age among Nigerian women [29] as well as among African women as general according to a recent meta-analysis [4].

The current study showed that women with high parity were at higher risk of obesity, which supports previous reports [4,29]. A possible explanation for this could be that most women do not return to their prepregnancy weight after giving birth, which might result in a higher weight in subsequent pregnancies. Alternatively, there may have been a cumulative weight gain throughout the previous pregnancies.

The prevalence of anemia in this report was higher than that previously observed in the same setting. Interestingly, while there were no significant group differences in the prevalence of anemia, hemoglobin level was significantly higher in obese women.

Our finding of higher hemoglobin levels among overweight and obese women compared with normal weight women accords with previous reports. Another study examining stillbirth found that higher BMI was associated with higher hemoglobin levels at the first antenatal visit [30]. Likewise, Kordas *et al*. [17] reported that overweight and obesity were associated with a lower likelihood of anemia among nonpregnant Colombian women in the reproductive age, and that overweight and obese women had higher estimates of hemoglobin.

On the contrary, in a small (23 women in each arm) study Crabb and Chamberlin reported significantly lower hemoglobin levels in obese patients when compared with their normal weight peers [31]. However, this study may differ as it was conducted in the second trimester while we investigated the first, and it was of small sample size.

The current study's results are also in contrast to the findings of a recent meta-analysis of 26 studies of nonpregnant women, including 13,393 overweight/obese individuals and 26,621 nonoverweight participants. The overweight/obese participants had lower serum iron concentrations than the nonoverweight and had a significantly increased risk of iron deficiency (OR: 1.31; 95% CI: 1.01–1.68) [32]. However, we have to acknowledge that serum ferritin is a better measure of iron status than hemoglobin level, so with just using the data on hemoglobin, the comparison will not precisely reflect the differences. Pregnant women may be different from nonpregnant women in this respect; accordingly, caution should be used in comparing results from these studies.

One of the limitations of the current study is that serum ferritin and C-reactive protein were not investigated. However, as according to WHO reports, the dominant type of anemia among pregnant women is iron deficiency [33], this limitation should be relatively minor. As an alternative to measuring serum ferritin, we relied on red cell distribution width, one of the components of the hemogram, as it is considered to be an inflammatory marker and a substitute for serum ferritin in diagnosing iron deficiency anemia [34]. Moreover, our previous studies showed no association between anemia and C-reactive protein [35]. Hemoglobinopathies and other factors that could have effects on hemoglobin level, such as smoking, were not investigated and thus this has to be considered a limitation of the current study. Smoking was not asked for because we feared loss of cooperation, as it is not socially acceptable among Sudanese women.

However, the current study's results showed that WBC count was significantly associated with overweight and obesity. This is in alignment with reports discussing the inflammatory nature of obesity [36,37]. Plausible explanations for the anemia among obese individuals were: dietary iron deficiency, increased demand for iron due to increased blood volume and physical inactivity and low-grade inflammation [6–8]. Furthermore, hepcidin (an iron-regulatory protein) and obesity-associated low-grade inflammation might play a pivotal role in the regulation of iron homeostasis [9,13–15]. Notably, anemia was present in approximately 50% of the current study's population. This high prevalence is in concordance with our previous finding of low usage of iron and folate supplementation among pregnant Sudanese women during their first trimester [38]. It is to be mentioned that BMI was taken in the early where prepregnancy BMI and weight gain during pregnancy might be difficult to get in our setting. Finally, the high hemoglobin level among obese women may be due to the nutritional status of obese group, for example intake of high iron foods.

## Conclusion

In the current study, obese women had higher WBC count and higher hemoglobin levels, and were more likely to be of older age and multiparae compared with nonobese women. This could reflect the inflammatory nature of obesity and warrants further study to better understand these findings.

## Future perspective

Given the increasing rate of obesity worldwide, where pregnant women are a vulnerable group, more effort is needed to explore the pathophysiology of obesity during pregnancy. The association between obesity and anemia (especially iron deficiency) might reflect the poor nutrition of obese women. Many biomarkers, for example ferritin and hepcidin, need to be investigated in depth. Furthermore, chemokines could have their influence in the pathophysiology of obesity. A longitudinal study investigating iron parameters and gestational diabetes and the effects of hemoglobin level on the maternal and perinatal outcome is needed.

**Executive summary**

- Obesity among pregnant women is a globally recognized, contentious public health problem, and can lead to adverse maternal and fetal outcomes.
- While there has been a great deal of research into the association between anemia/hemoglobin levels and obesity among nonpregnant women, there are no published data on hemoglobin levels and obesity among pregnant women.
- The objective of this study was to investigate the prevalence of obesity and the association between obesity, hemoglobin levels and white blood cells (WBCs) count among a population of Sudanese women in their early pregnancy.
- A cross-sectional study was conducted at Saad Abualila Hospital (Khartoum, Sudan) during the period of January–April 2014.
- Sociodemographic, medical and obstetric data were collected using questionnaires. Weight and height were determined, and BMI was calculated.
- The complete maternal hemogram was measured for all participants.
- One-way analysis of variance and χ^2^ tests were used to compare the normally distributed continuous variables and proportions between BMI groups.
- A multinomial logistic regression was conducted where the BMI group was the dependent variable and sociodemographic parameters, hemoglobin levels and WBC count were the independent variables.
- p < 0.05 was considered statistically significant at two-sided test.
- The mean (standard deviation) of the age, parity and gestational age was 27.8 (5.5) years, 0.9 (1.2) and 10.5 (3.1) weeks, respectively.
- There were 15 (4.4%), 95 (28.1%), 127 (37.6) and 101 (29.9%) women who were underweight (<18.5 kg/m^2^), normal weight (18.5–24.9 kg/m^2^), overweight (25–29.9 kg/m^2^) and obese (≥30 kg/m^2^), respectively.
- While there was no significant difference in education level, residence and occupation, age and parity, hemoglobin levels and WBC count were significantly higher in obese than nonobese groups and were higher with increasing BMI.
- In the multinomial analyses, parity was higher with increasing BMI for the overweight and obese women. Hemoglobin levels were higher with increasing BMI, with odds ratios (95% CI) of 0.6 (0.4–1.0), 1.4 (1.1–1.8) and 1.3 (1.06–1.8), for underweight, overweight and obese women, respectively.
- The odds ratios for WBC count in the underweight and obese women compared with normal weight women were 0.6 (0.4–0.9) and 1.1 (0.9–1.3), respectively.
- Previous study reported a significantly lower second trimester hemoglobin levels in obese patients when compared with their normal weight peers.
- On other hand another study examining stillbirth found that higher BMI was associated with higher hemoglobin levels at the first antenatal visit.
- A longitudinal study to investigate iron parameters and gestational diabetes and other adverse effects of hemoglobin level on the maternal and perinatal outcome is needed.
